# Rash Action of the House of Delegates

**Published:** 1905-05

**Authors:** 


### TEXAS MEDICAL JOURNAL.

AUSTIN, TEXAS.

A MONTHLY JOURNAL OF MEDICINE AND SURGERY.

EDITED AND PUBLISHED BY
F. E. DANIEL, M. D.
Mrs. F. E. DANIEL, Business Manager.

Published Monthly at Austin, Texas. Subscription price $1.00 a year in advance.

Eastern Representative: John Guy Monihan, St. Paul Building, 220 Broadway New York City.

Official organ of the West Texas Medical Association, the Houston District Medical Association, the Austin District Medical Society, the Brazos Valley Medical Association, the Galveston County Medical Society, and several others.

### RASH ACTION OF THE HOUSE OFDELEGATES.

The State Medical Association, as will be seen from the report of the reecnt meeting, resolved to establish a monthly journal, in which to publish its proceedings, instead of a yearly volume of transactions. The journal will contain, also, news, editorials, book reviews, etc., as other journals do, and it is to be edited by the secretary, who is to be paid a salary.

We seriously question the wisdom of the step at this time. The action was precipitate and not well considered except by the publishing committee, who submitted figures to show that, by the aid of advertising they expect to get, the journal can be made to cost the Association less than the yearly volume of transactions. The estimate was made upon a 48-page quarto, of which 16 pages are to be devoted to advertisements when they are secured.

Such size publication will be totally inadequate to the work, and will not contain the papers alone which were read at the meeting. There were 120 papers. These, with the discussions, the minutes, the addresses, the lists of officers, committees, roll of members, editorials, book notices, etc., will require a journal five times the size of the one estimated on. And, notwithstanding the small size, the first item on the submitted estimate ($2250 for *printing alone*), exceeded the total cost of last year's handsome volume

(2550 copies of 650 pages each) of Transactions, post paid, delivered.

The success, even partly, of the venture, depends, primarily, upon the committee securing sixteen pages of advertising, which they estimate at $2400; and secondly, upon the regular and prompt payment of dues by 2500 members. In both these respects I believe the committee will be disappointed. The venture involves much risk, and threatens disaster in case of failure of either or both contingencies. Besides, *only $1 of the $2 annual dues is to be appropriated to the journal enterprise.* The expenses of the Association outside of the journal, averaged in 1904, over $150 a month ($1800 a year), and much of the dues of 1905 were used to pay the debts of 1904.

I did all in my power to induce the House of Delegates to have Dr. Wilson (ex-president and father of the journal idea) to name four other ex-presidents to serve with him as a committee to investigate and report at next meeting how large a journal will be required and what it will cost, and in the meantime to get a consensus of the wishes of our large membership as to changing to a journal or continuing the yearly volume; but my caution to "look before you leap"; "be sure you can do it before you undertake it," was without avail. The proposition was railroaded through the House with a whiz. Already I have heard much complaint from the older members and the rank and file that they were given no voice in the matter, and they are dissatisfied. The yearly volume is valuable and a permanent record and an ornament to the library. The set for twenty years is complete. We now make a gap, as we did in 1878, when journalizing was tried six years, and abandoned. I fear we will all regret the experiment before next meeting. However, we will see.

Let us put it this way: *If* 2500 members pay $2 dues each, the income will be $5000 a year. Of this, $1 from each payment is available for all journal purposes, $2500; deduct editor's salary, $1200, and we have $1300 a year with which to pay printing, $2250, office expenses, postage, stationery and incidentals, say $750. There is a shortage of, say $1700; and this is the estimate on forty-eight pages, sixteen of which are for ads to be secured. That leaves 32 pages a month for proceedings, papers, addresses, discussions, editorials, news and miscellaney, abstracts, reports of committees, book notices, etc. That will give us, *in a year* $12 \times 32 = 384$ pages, a year, available for all reading as above; and each page, it is said, is equal to two and one-half pages the size of the annual volume of Transactions. We thus have available for all journal purposes, $384 \times 2\frac{1}{2} = 960$, pages, *a year* in which to publish all that pertains to the proceedings, addresses, papers, discussions, editorials, book reviews, etc. The 1904 volume of Transactions alone made 648 pages. This year we will have, of *papers alone,* not less than 1000 pages. It is a physical impossibility to publish the papers alone in twelve months, "which is why I remark"—it will take a 32-page journal two years to give the members the work done at the Houston meeting, and two years' income to pay for it, in addition to those ads upon which the committee so confidently rely.

Suppose that in the *twelve* months all the papers and other matter *can* be published, somebody's paper will be lost, of course, and there'll be kicking, if not curses loud and deep. How would you like to have your paper appear three months after next meeting? Will a member carefully file his journals for twelve months, and then send them by express to Austin, Dallas, or elsewhere to be bound, paying expressage each way and $2 a volume for binding? Not much. Not one out of a hundred will do it, and if one should do so he will find that the twelve issues will make two large volumes, and the total cost to him will be about $7, instead of the $2 for his annual dues, which entitled him to the handsome volume of Transactions.

I believe the House has made a great mistake. They will find, when too late, that members, deprived of the yearly volume and who seldom go to the annual meetings, will simply drop out. I have heard many say so, already. Now, lest I be misunderstood,—having a journal of my own,—I repeat here what I said in the House: "I don't want to edit a State Association journal; I don't want the "Red Back" made official organ, under control of a board of trustees, under any circumstances. Now, with this understanding, let us discuss the proposition soberly and with judgment."

One of the "arguments" for a journal, submitted by the publication committee, was that "there is no journal in the State that ever publishes anything about the Association or its meetings or legisative matters and bills." This is an unjust impeachment of the "Red Back." Let this issue alone refute it. All my readers know that for twenty years the "Red Back" has "made a specialty" of these matters and a copy has been sent *free* to many members who couldn't see the propriety of subscribing $1 a year to get it. A copy containing all the news and bills was sent free to the secretary and the president of every county medical society in the State, many of whom were not subscribers.

After the action of the House above related, new subscribers tumbled in, scores of them, and the "Red Back" is on a new boom.

